# Development and Application of a New Grey Dynamic Hierarchy Analysis System (GDHAS) for Evaluating Urban Ecological Security

**DOI:** 10.3390/ijerph10052084

**Published:** 2013-05-21

**Authors:** Chaofeng Shao, Xiaogang Tian, Yang Guan, Meiting Ju, Qiang Xie

**Affiliations:** 1 College of Environmental Science and Engineering, Nankai University, Tianjin 300071, China; E-Mails: guanyang@mail.nankai.edu.cn (Y.G.); jumeit@nankai.edu.cn (M.J.); 2 Sichuan Institute of Science and Technology of Environmental Protection, Chengdu 610041, China; E-Mails: txg20032003@yahoo.com.cn (X.T.); fzili@163.com (Q.X.)

**Keywords:** indicator selection, grey dynamic hierarchy analytic system (GDHAS), driving force-pressure-state-impact-response (DPSIR), urban eco-security assessment, Tianjin city

## Abstract

Selecting indicators based on the characteristics and development trends of a given study area is essential for building a framework for assessing urban ecological security. However, few studies have focused on how to select the representative indicators systematically, and quantitative research is lacking. We developed an innovative quantitative modeling approach called the grey dynamic hierarchy analytic system (GDHAS) for both the procedures of indicator selection and quantitative assessment of urban ecological security. Next, a systematic methodology based on the GDHAS is developed to assess urban ecological security comprehensively and dynamically. This assessment includes indicator selection, driving force-pressure-state-impact-response (DPSIR) framework building, and quantitative evaluation. We applied this systematic methodology to assess the urban ecological security of Tianjin, which is a typical coastal super megalopolis and the industry base in China. This case study highlights the key features of our approach. First, 39 representative indicators are selected for the evaluation index system from 62 alternative ones available through the GDHAS. Second, the DPSIR framework is established based on the indicators selected, and the quantitative assessment of the eco-security of Tianjin is conducted. The results illustrate the following: urban ecological security of Tianjin in 2008 was in alert level but not very stable; the driving force and pressure subsystems were in good condition, but the eco-security levels of the remainder of the subsystems were relatively low; the pressure subsystem was the key to urban ecological security; and 10 indicators are defined as the key indicators for five subsystems. These results can be used as the basis for urban eco-environmental management.

## 1. Introduction

With the rapid development of society and economy throughout the World, ecological and environmental problems have become increasingly serious threats to our living environment. In the middle of the twentieth century, concerns began to mount over problems caused by ecological degradation, and ecological security has become a common interest for many countries and organizations [1]. Ecological security, a concept first proposed by the United States government [2], has been linked with national security, economic security, and human well-being [3]. In addition, urban ecological security, a spatial concept, is the possibility of variation trends, guaranteed by a complex system of humans and the environment within an urban region, which prevents potential risks to the human ecological security space caused by natural and human activities or a combination thereof. The urban ecological security to be discussed in this paper includes natural, economic, and social factors of an urban area, focusing on interactions between economic development and environmental protection. As human activity rapidly continues to encroach into natural areas, the importance of the effective assessment of urban ecological security becomes more imperative. As centers of socio-economic production and human consumption, cities play a driving role in the development of regional, national, and even international economics [4]. However, the urban ecosystem is fragile and unstable, with a high consumption of energy and material and low amounts of natural resources. Environmental pollution and ecological degradation impact cities undergoing rapid urbanization. The assessment of urban ecological security is necessary to cope with these problems and provide the basis for urban planning and management. Thus, methods to assess urban ecological security are an absolute requirement for the sustainable development of a city. 

Several conceptual frameworks and mathematical modeling approaches have been employed in this field, and the process of urban ecological security assessment generally contains two steps: (i) establishing the indicator system for assessing urban ecological security and (ii) applying the quantitative models to process the indicator data and define the status of urban ecological security.

In the first step, the theories of the ecological footprint [5], energy analysis [6], and landscape ecological analysis [7,8] have been applied to establish the indicator system. However, because the assessment is complex and incorporates numerous social, economic, environmental, and ecological factors [9], the framework based on these theories covers only a portion of the factors required for urban ecological security assessment, and the hierarchies and integrity of the indicators often are ignored. Thus, a conceptual framework on which to build an indicator system that can integrally describe all aspects of urban ecological security with clear hierarchies is needed. The pressure-state-response (PSR) model, which was developed based on Statistics Canada’s ‘‘Stress-Response Environmental Statistical System’’ (SRESS) [10], and its derivative models, such as the DPSRC (driving force-pressure-state-response-control) model [11] and the driver-pressure-state-impact-response (DPSIR) model [12], are now widely applied in establishing the indicator system for ecological and environmental assessments [13,14]. These models can clearly reflect the causality of the indicators [15] and describe urban ecological security from multiple relative aspects.

In the second step of the assessment, several quantitative modeling approaches, including the analytic hierarchy process [7], Bayesian belief networks [12], back propagation (BP) neural network channels [16], and the attribute theory model [17,18] are combined with the PSR models to define the urban ecological security status of the city under study. The results of these composite methodologies can be further improved by using sets of TM/ETM images and the GIS technique to assess regional/urban ecological security [7,15,19].

Before constructing the framework based on the PSR and its derivative models in the first step, the indicators of urban ecological security must be selected. However, this process often was ignored in previous research, and few studies have focused on methods of indicator selection. The lack of consideration of the relationship between subsystems and their indicators in urban ecological security assessment has hindered the efforts of conventional modeling approaches in this field. However, few modeling approaches employed in the second step performed very well in analyzing the relevance, hierarchy, and dynamics of the indicators simultaneously. Therefore, developing and applying quantitative methods to select the indicators of ecological security for the whole framework before beginning the first step is crucial to the assessment of urban ecological security.

The goal of this study is to develop a new systematic methodology for urban ecological security assessment and take a typical Chinese city as an example to test the practicality and operability of this systematic methodology. This systematic methodology contains the following three steps: (1) dynamic selection of indicators; (2) establishment of the indicator system; and (3) quantitative assessment of ecological security that facilitates the entire process of urban ecological security assessment dynamically according to the characteristics of the given study area. This innovative systematic methodology is based on the analytic hierarchy process (AHP) approach [20], grey target theory (GTT) [21], the DPSIR model, and the fuzzy assessment model [22,23,24], and the details are described in Section 2. Therein, the modeling approach for indicator selection, the dynamic indicator system, and the quantitative method for defining the weights of the indicators are outlined; furthermore, the objectives of the computational studies are described in detail. Section 2 also introduces the programs of the model and provides a brief overview of its development and features. Section 3 describes the application of the model to a typical Chinese city (Tianjin), and final comments and conclusions are provided in Section 4.

## 2. Methodologies

### 2.1. Background and Requirements

Each urban area has factors which may affect its ecological security and as the corresponding processes often change with dynamic variations of the factors, appropriate indicators must be selected based on their impacts on urban ecological security and on the characteristics of the particular urban area under study. This step must be performed before building the assessment framework. Thus, a quantitative modeling approach that can define the importance of indicators dynamically and also handle multiple criteria and objectives plays a crucial role in the selection process. During the process of indicator selection, a number of uncertain factors exist, data from different perspectives (e.g., society, economy, environment, and ecology) must be integrated, and the dynamic variation of the indicators needs to be reflected. Because of the complex conditions, several traditional approaches are rarely used in this field.

The AHP model facilitates characterization of the differences that exist between one alternative and another in the resultant priority vector. It is widely used in establishing the assessment framework with PSR and its derivative models [7,15], and it allows all of the relative respects including society, economy, environment and ecology to be considered in indicator selection. However, it depends excessively on the subjective weights of each of the performance indices, and the complicated interrelationships among multiple performance indices are ignored [17].

The grey system theory developed by Deng in the 1980s [21] has been successfully applied in recent years to engineering prediction and control, social and economic system management, and environmental system decision making [25]. It is useful for dealing with poor, incomplete, and uncertain information. GTT is one of the major modeling approaches of the grey system theory. And the GTT can be used to effectively identify the complicated interrelationships among multiple performance characteristics through the optimization of grey relational grades. However, this model can not be applied to simulate the comprehensive procedures with several hierarchies of indicator selecting.

The PSR model widely used in ecological and environmental assessment [10,13] can be used to classify alternative indicators, build the indicator system, and reflect the causality among the indicators in each level of the framework. However, the PSR model has its own inherent limitations: Its focus on isolating pressures, states, and responses tends to provide a static representation of eco-security and ignores the significant dynamic processes that constitute the interactions among these components.

The fuzzy assessment model that is widely applied in the engineering, environment, and economy fields [22,23,24] can be applied to standardize the indicators and define the level of eco-security for the indicators based on the weights of the indicators in the last step of the modeling system for assessment [1,15].

To take advantage of the useful parts of the basic methods, including AHP, GTT, DPSIR and the fuzzy assessment model, for our particular purpose, we developed a grey dynamic hierarchy analysis system (GDHAS) model that integrates GTT into the AHP; this integration maintains the basic structure of the traditional AHP but replaces the process of expert group judgment with the GTT. Our innovative modeling approach can be combined with the DPSIR framework and the fuzzy assessment model to generate a systematic methodology to select indicators, build the indicator system, and define the level of ecological security. This methodology eliminates subjective errors and addresses the entire process of the assessment dynamically.

**Figure 1:**
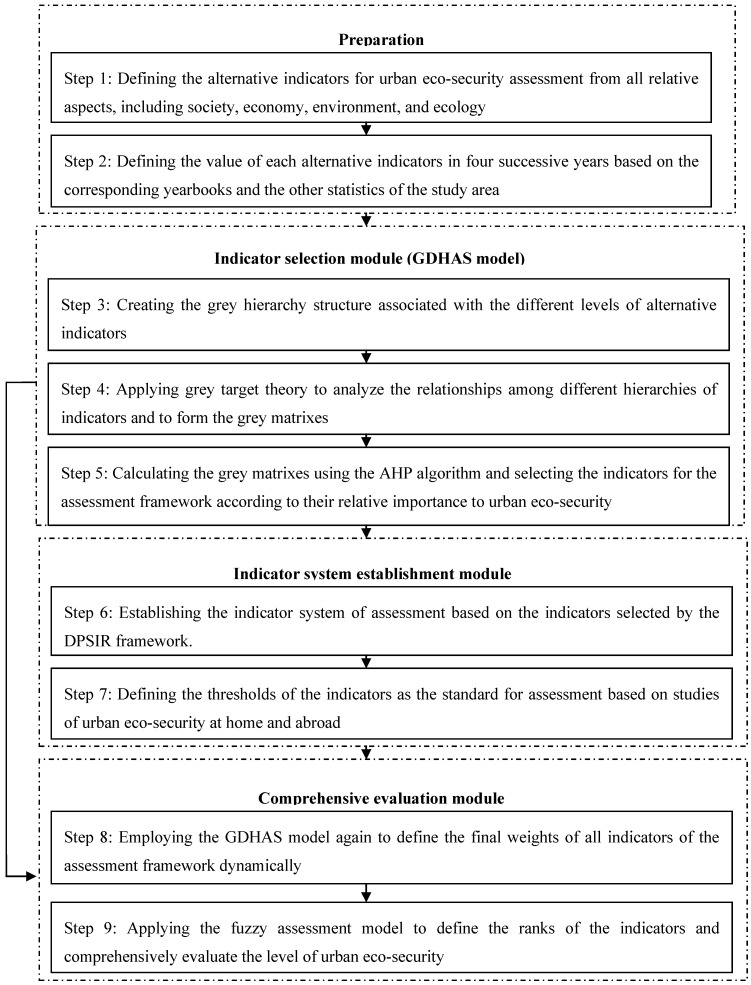
Flow chart showing the systematic methodology for urban eco-security assessment.

### 2.2. Structure of the Methodology

The systematic methodology of urban ecological security assessment contains three modules: indicator selection, establishment of the indicator system, and quantitative evaluation (Figure 1). In the indicator selection module, the GDHAS model can be used to define the weights of the indicators in different levels with the GTT process, form the grey matrices and link the grey matrices of all the hierarchies by employing the AHP. During this process, the final weights of the indicators of urban ecological security can be obtained by calculating the grey matrices, and the indicators for establishing the DPSIR framework can be selected based on their significance to urban ecological security. In the module for establishing the indicator system, the DPSIR framework is applied to build the indicator system for urban ecological security assessment based on the indicators selected in the first module. Finally, in the quantitative evaluation module, the GDHAS model is used again in conjunction with the fuzzy assessment model to define the final weights of the indicators and the level of urban ecological security based on the assessment framework. Thus, urban ecological security can be assessed from multiple corresponding aspects dynamically according to the characteristics of the study area.

Figure 1 shows that our systematic methodology for urban ecological security assessment can not only select the indicators dynamically and establish the assessment framework according to the characteristics of the study area but also assess urban ecological security comprehensively through the quantitative modeling approach. The results then can be used as the basis for decision making to manage the ecology, environment, society, and economy of the urban area.

### 2.3. Indicator Selection

As different urban areas have different factors which affect their ecological security, selecting the representative indicators which can indicate the urban ecological security of a given area plays a significant role in the assessment of urban ecological security. Thus, to select the proper indicators quantitatively based on their dynamic variation, we developed the GDHAS to define the importance of the alternative indicators for the assessment of urban ecological security.

#### 2.3.1 Alternative Indicators

Ecological security theory states that the internal structure of ecosystems is logical and stable and that the function of ecosystems is positive and tends towards health [26]. The assessment of urban ecosystem health focuses more specifically on the ecological state and functions [27], which can be viewed as an important part of urban ecological security assessment. Considering the different viewpoints of urban ecological security and eco-health, several indicators have been developed and studied (Table 1), including aspects of ecology, environment, economy, and society [15,28,29,30]. Thus, we can define alternative indicators, which may represent the characteristics of the study area, for urban ecological security assessment by summarizing previous studies of urban ecological security assessment and urban eco-health assessment and by discerning the characteristics of the given study area.

**Table 1:** Main related indicators for the urban ecological security assessment.

Person and reference	Indicators
The World Health Organization: (1) The Building from Healthy Cities Project proposed 79 healthy urban ecosystem indicators in 1996 [31].	Internal character
	External performance
	Progress
	Management and monitoring
	Providing service
	Budget and finance
	Community service
(2) Takano and Nakamura further developed 459 indicators of a healthy urban ecosystem in 1998 [32].	Human health
	Urban infrastructure
	Environmental quality
	Human housing and living environment
	Community’s role and action
	Living pattern and prevention performance
	Health care and environmental sanitation service
	Education
	Employment and industry
	Income and domestic consumption
	Local economic and demographic statistics
(3) Guo *et al.* used the classic framework from established urban eco-healthy indicators using 24 factors [33].	Vigor: GDP *per capita*
	Organizational structure: the third industry ratio
	Resilience: treatment rate of urban domestic sewage
	Ecosystem services maintenance: comprehensive
	environmental quality index
	Population health: mean human life time
(4) Yang *et al.* established an indicator system within the DPSRC (driving force-pressure-state-response-control) framework [34].	Environment: environmental quality and solid waste utilization
	Resource: land, water, and atmosphere resources *etc.*
	Society: population intelligence and average literacy
	Economy: public services facilities, science and technology input index
(5) Hu *et al.* proposed indices to measure the gap between the urban developmental status quo of each factor and certain development objectives [35].	Distance index
	Coordination index
(6) Shi *et al.* developed an indicator system to assess urban eco-security mainly from resources and the eco-environment [36].	Resources security index
	Pollution index
	Ecology index
(7) Su *et al.* established a biophysical urban eco-security indicator system using 17 related energy-based indices [37].	Vigor: energy density
	Structure: energy diversity index
	Resilience: carrying capacity density based on renewable energy
	Ecosystem service: environmental loading ratio
	Population health: energy investment ratio
(8) Liu *et al.* (2009) developed an energy-based urban eco-security indicator that integrates vigor, organizational structure, resilience, and function maintenance [38].	Net energy yield ratio
	Environmental loading ratio
	Energy exchange ratio
	Energy density

#### 2.3.2. Selection Hierarchy System

**Figure 2:**
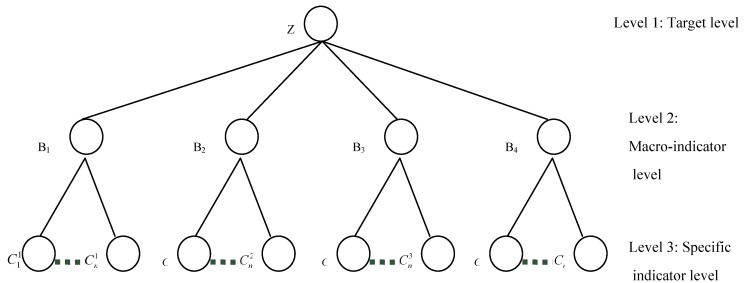
Grey hierarchy system used for indicator selection.

Selecting the key indicators for assessing the ecological security of an urban area involves analyzing the ecology, environment, society, and economy of the area, with each category containing several indicators with hierarchical characteristics. Figure 2 shows a grey hierarchy system for the key indicator selection to assess urban ecological security with three hierarchies. The overall objective of the urban ecological security assessment lies at the top of the hierarchy (Level 1), and the macro-indicators (MIs) and the specific indicators (SIs), which represent grey sequences, lie at descending levels of this hierarchy (Levels 2 and 3, respectively) (Table 2). Five MIs (driving force, pressure, state, impact, and response) constitute the second level. The SIs, which can be classified into five groups corresponding to the MIs, are at the bottom level.

**Table 2:** Main related indicators for the urban ecological security assessment.

Indicator type	Indicator explanation
macro-indicators (MIs)	driving force
	pressure
	state
	impact
	response
specific indicators (SIs)	62 alternative indicators

The evaluation of the ecological security of a given urban area involves synthesizing a considerable number of MIs and SIs with their corresponding standards so that the urban ecological security assessment of a given urban area lies at the top of the grey hierarchy as the target level. The MIs, including ecology, environment, society, and economy, describe the macro-effects on urban ecological security and reflect the effects of the various types of SIs. The SIs can be classified into four groups that correspond to the MIs (e.g., the ratio of vegetation cover in an established area corresponds to ecology, the discharge intensity of the chemical oxygen demand (COD) corresponds to the environment, the number of advanced degrees per 10,000 people corresponds to society, and GDP *per capita* corresponds to the economy). The MI and SI levels in Figure 2 show the corresponding indicators to be compared and evaluated. For a given urban area, we take the local typical SIs into account, and the grey hierarchy system can be modified according to the reality of that particular study area.

Figure 2 includes multiple indicators of urban ecological security, and a three-level hierarchy structure is built to organize these indicators, which is a special design which can reduce the transmission of errors during the calculation procedures.

#### 2.3.3. Grey Target Theory (GTT) Procedure

In the GTT procedure, indicators of the single-response grey relational grades are simplified from the complex multiple-response indices, which consist of the specific indicators and macro-indicators. The application of this procedure is conducive to understand the relationship between the key indicator and other related indicators in a given system. The function of GTT is to define the grey relational grades of both SIs and MIs in a given urban area, which is described as below.

First, in this grey hierarchy system, the following analysis is built on two grey influence spaces. One, denoted as *w_i_*^(1)^(*k_1_*), is developed by the original grey sequences of the macro-indicators (MI), and the other, denoted as *w_i_*^(2)^(*k_2_*), is developed by the original grey sequences of the specific indicators (SI). They are the objects of the grey relational analysis, and these two matrices represent the series of MI and SI data, respectively, for the study urban area in the ith year.

Second, in the SI level, the condition of urban ecological security presents positive or negative variations of the indicators, which are decided by how these indicators work while assessing urban ecological security. Thus, different grey sequences of indicators vary from the poles. Some SIs, such as the ratio of forest cover and the proportion of environmental protection investment to the GDP, fall into one category as the level of urban ecological security and the values of such indicators present a positive correlation. In this case, the response grey sequences are viewed as the minimum pole sequences. In contrast, SIs, such as the discharge intensity of COD and SO_2_, that lead to ecological degradation represent a negative correlation with urban ecological security, which are then categorized as the maximum pole indicators. At the MI level, the poles of the indicators, which present social, economic, environmental and ecological impacts on urban ecological security, rely on the comprehensive role of the corresponding SIs. Consequently, the sequences of both MIs and SIs shall be from a consistent pole using the T algorithm.

Third, based on the pole transformation and the equation presented below, the grey relational coefficient of the MI and SI can be defined as *ã*^(1)^(*x_i_*(0),*x_i_*(*k_1_*)) and *ã*^(2)^(*x_i_*(0),*x_i_*(*k_2_*)) respectively:

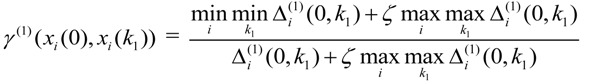
(1)

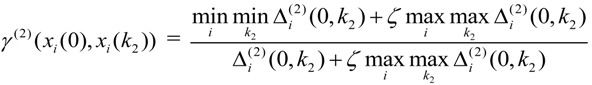
(2)

In the equations shown above, 
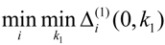
 and 
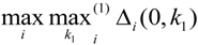
 represent the minimum and maximum distances, respectively, for all MIs in the response sequences, and 
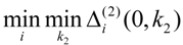
 and 
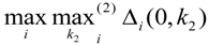
 represent the minimum and maximum distances, respectively, for all SIs in the response sequences. The distinguishing coefficient *ζ* is defined within the range 0 ≤ *ζ* ≤ 1. In general, *ζ* has a value of 0.5 (*ζ* = 0.5) according to the least information principle [21]. Then, the grey relational grades of the MIs and SIs are derived, respectively, as follows:

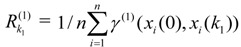
(3)

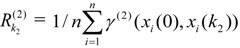
(4)

As represented above, *R_K1_*^(1)^ in the equation denotes the effect of the k_1_^st^ MI on urban ecological security and *R_K2_*^(2)^ denotes the effect of k_2_^nd^ SI on the corresponding MI during the same time period. A higher grey relational grade indicates that the standard contribution sequence is more similar to the grey contribution sequence, which means that the response MI or SI has a greater impact on the urban ecological security assessment.

#### 2.3.4. Application of the Analytic Hierarchy Process (AHP)

The AHP links the grey relational grades of the MIs and SIs, which are obtained through the GTT approach via the hierarchy calculation of the MI and SI grey matrices. It is also used to define the final weights of the SIs in the urban area of study. Compared with the traditional AHP, this method with the application of the grey relational grades is presented as more objective and accurate than the original approach, which was, to a large extent, influenced by the experts’ views. Because the grey matrices of the MIs and SIs for the hierarchy analysis are developed on the basis of grey relational grades rather than subjective scores given by experts, the grey matrix of the MIs can be developed, where *p_xx_* = 1 (on the diagonal) and *p_xy_* = 1/*p_yx_*.

The four grey matrices of the SIs for ecology, environment, society, and economy can be developed in a similar way. The corresponding eigenvalues of the SIs in parts of these four matrices can be obtained in accordance with each MI; as a consequence, the vectors of the SI level can be formed, which are expressed as:

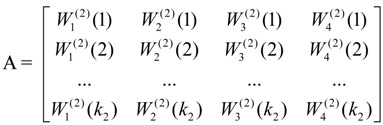
(5)

Through the consistency test [39] of the eigenvalue of matrices A and G, the integrated weights of the SIs, which reveal the relationship between the MI schemes and the SI schemes for the overall objective, are derived from the following:

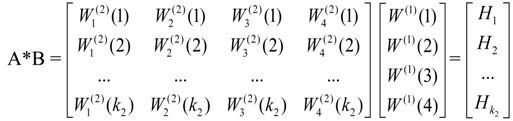
(6)

*H_k2_* in the matrix above represents the impact of the k_2_^nd^ SI on the urban ecological security of the study area. Basically, the larger the final weights of the SI are, the greater the effects on urban ecological security will be. If *H_k2_* ≥ 1/*N*, with N representing the number of alternative indicators, the corresponding SI can be viewed as a key indicator in the urban ecological security assessment. In contrast, if *H_k2_* < 1/*N*, the corresponding SI will be filtered out. Thus, based on their final weights, the key indicators with significant impacts on the urban ecological security can be distinguished and further applied to establish an assessment framework. Meanwhile, with suggestions given by local experts, a more optimized group of indicators will be presented, especially in the determination of the low weight value of the indicators compared with 1/*N*.

### 2.4. Construction of the Indicator System

#### 2.4.1. Application of the Driving Force-Pressure-State-Impact-Response (DPSIR) Model

**Figure 3:**
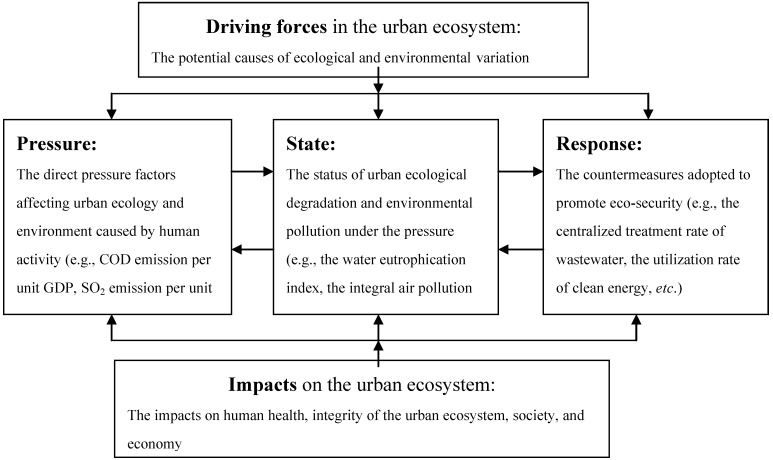
The DPSIR framework for the urban eco-security assessment.

The DPSIR model that is derived from the traditional PSR model is employed as the foundation of the indicator system for assessing urban ecological security based on the indicators selected in the first module. The indicators can be classified into five groups—driving forces, pressure, state, impact, and response—according to the causality among them, and they are organized within the DPSIR framework as shown in Figure 3.

Figure 3 shows the procedure for constructing the indicator system for assessing urban ecological security which is based on the DPSIR framework and the causality of the five components.

#### 2.4.2. The Thresholds for the Assessment

Appropriately defining the thresholds of the indicators plays a crucial role in the urban ecological security assessment. As a requirement of the assessment, each indicator is assigned for two states: safe or unsafe. Based on previous studies that analyzed the standards and thresholds of various indicators [16,23,40] and on suggestions from experts, the thresholds of the indicators can be defined using one of three methods: (1) when a threshold is known, *i.e.*, the warning values and optimum values recognized internationally are available, it is used with a 20% fluctuation; (2) when an indicator has an associated legislative value, above which its state would be in breach of law, this value can be used as the threshold; however, this method is seldom practical as such values are rarely significant to ecosystem functioning [12]; and (3) the median value is the most appropriate threshold in most cases because it allows the greatest possible overlap between linked datasets.

### 2.5. Comprehensive Evaluation

#### 2.5.1. Defining the Weights of the Indicators

In this module, the GDHAS modeling approach developed in the first module is employed once again to define the final weights of the indicators of the assessment framework dynamically. The procedure of defining the weights of the indicators is shown as follows:

First, the grey hierarchy system for dynamic analysis can be established according to the DPSIR assessment framework mentioned above (Figure 3). Compared with the selection hierarchy system (Figure 2), the urban ecological security assessment is still the overall objective of the hierarchy at the top level. Five MIs (driving force, pressure, state, impact, and response) constitute the second level. The SIs, which can be classified into five groups corresponding to the MIs, are at the bottom level.

Second, the GTT procedure is applied to determine the grey relational grades of the MIs in the second level and of the SIs in the bottom level. The SI and MI data for four successive years are then input into the dynamic analysis.

Third, the grey relational grades of the five MIs and the corresponding SIs are used to form the grey matrices, and the final weights of the MIs and SIs can be calculated using the AHP algorithm.

In this way, the SIs that have the greatest impacts on urban ecological security can be defined, and the final weights of the MIs and SIs can then be used to determine the level of urban ecological security.

#### 2.5.2. Application of the Fuzzy Assessment Model

The fuzzy assessment model is used to standardize the SIs so that they can be compared with each other. The values of the SIs are normalized in the interval [0,1] following the standardization procedure. However, because different SIs have different poles in the urban ecological security assessment, the fuzzy assessment model can be described under the following conditions.

Standardization of the SIs with positive poles:

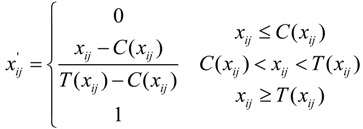
(7)

Standardization of the SIs with negative poles:

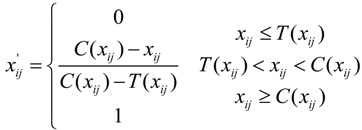
(8)
where *x_ij_* and *x_ij_*' represent the original value and the standardized value of the ith SI belonging to the jth MI, respectively, and *T*(*x_ij_*)and *C*(*x_ij_*) are the higher and lower limits, respectively, of the corresponding SI associated with urban ecological security. The ecological security scores of the SIs can be calculated using Equation (9) based on the SI’s final weight and standardized value. The score of each MI then can be obtained by accumulating the corresponding scores of the SIs with Equation (10), where *w_ij_* is the weight of the corresponding SI and *P_ij_* and *Q_i_* are the ecological security scores of the SI and MI, respectively:

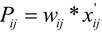
(9)

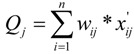
(10)

Finally, the ecological security of the entire urban area can be scored based on the ecological security score of the SI and MI, and then the comprehensive assessment of urban ecological security can be made dynamically from the perspectives of both a single indicator and the overall objective.

## 3. Case Study

### 3.1. The Study Area

Because the urbanization process is concentrated into large and complex urban and economic entities, city-centered regional development is popular in China [6]. However, such development has considerable impacts on urban ecological security. In response, China’s government has taken a series of actions to reduce these impacts and to ensure urban ecological security. One of the most significant measures has been the planning and construction of eco-cities throughout China.

**Figure 4:**
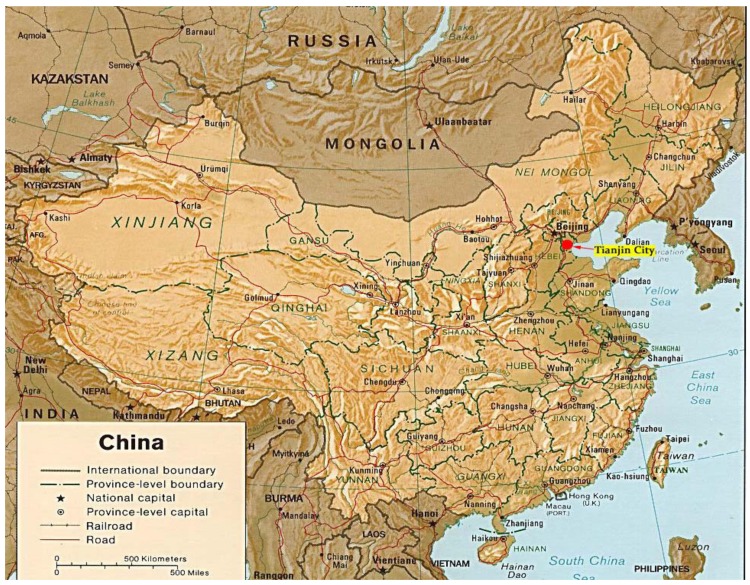
Map showing the location of Tianjin city in China.

Tianjin is one of the four municipalities in China. It is situated in the eastern part of the North China Plain, covers an area of 11,300 km^2^, and has a population of 6 million. Tianjin is one of the biggest industrial centers in China, with machinery, electronics, textiles, and chemicals and is a key sea port for Beijing, North China, and Northwest China (Figure 4). Therefore, Tianjin is a typical coastal super megalopolis that is undergoing rapid development to implement the planning and construction of an eco-city in China.

In this case study, the systematic methodology described in Section 2 will be applied to assess the urban ecological security of Tianjin City comprehensively and dynamically. The results can be used as the basis for the planning and construction of the eco-city of Tianjin.

### 3.2. Application of the Methodology and Results

Two steps were involved in defining the alternative indicators for selection. First, the related policies and requirements of the Chinese government, including the indicator system of the eco-city and the environmental model city, were taken into consideration to determine the alternative indicators. Previous studies on urban ecological security assessment worldwide were analyzed, and the characteristics of Tianjin City were identified in this step. Second, we consulted 18 experts on ecology, environment, and circular economy from the Chinese research community in Tianjin (e.g., Nankai University, Tianjin University, and the Tianjin Institute of Science and Technology of Environmental Protection) to adjust the alternative indicators so that they would be both scientifically sound and practically applicable (Table 3). Finally, the GDHAS modeling approach was used to analyze the importance of 62 alternative indicators (Table 3) to the urban ecological security of Tianjin City. Indicators with weights >1/N were used to build the assessment framework, as shown in Table 3.

Table 3 lists the 39 indicators covering the economic, environmental, ecological, and social sectors that were selected. The DPSIR model then was applied to establish the assessment framework based on these indicators: four indicators constitute the driving force system, 11 indicators constitute the pressure system, nine indicators constitute the state system, six indicators constitute the impact system, and nine indicators constitute the response system (Table 4).

**Table 3:** Candidate indicators and selection results.

Dimension of the indicator	Indicator name		Unit	Weight for selection	Selection	Source of data
Economy	GDP *per capita*		$/person	0.013768		Statistical data ^a^
	*Per capita* annual government revenue		$/person	0.011788		Statistical data ^a^
	*Per capita* disposable income of urban households		$/person	0.016788	√	Statistical data ^a^
	Energy consumption of GDP		tons of SCE/$	0.016857	√	Statistical data ^a^
	Ratio of clean energy consumption		%	0.017532	√	Statistical data ^a^
	Water consumption of GDP		m^3^/$	0.019197	√	Statistical data ^a^
	Fresh water consumption of value-added industry		m^3^/$	0.024145	√	Statistical data ^a^
	Coefficient of effective utilization of agriculture irrigation water		-	0.016132	√	Eco-city planning
	Proportion of tertiary industry production to GDP		%	0.016505		Statistical data ^a^
	Rate of enterprises in scale certified by ISO-14000		%	0.013497		Statistical data ^a^
	Proportion of actual investment of protecting the environment in projects		%	0.019258	√	Statistical data ^a^
	Household consumption		$/person	0.012476		Statistical data ^a^
	Value-added industry *per capita*		$/person	0.016321	√	Statistical data ^a^
	Ratio of value added to gross industrial output value		%	0.015204		Statistical data ^a^
	Energy consumption of value added in industry		tons of SCE/$	0.017057	√	Statistical data ^a^
Society	Population density		person/ km^2^	0.016783	√	Statistical data ^a^
	Natural growth rate		%	0.013511		Statistical data ^a^
	Rate of public satisfaction with environment		%	0.01646	√	Eco-city planning ^c^
	urbanization level		%	0.014823		Statistical data ^a^
	Popularity rate of central heating		%	0.016291	√	Statistical data ^a^
	Perfectness ratio of lifeline systems in the city		%	0.01676	√	Eco-city planning ^c^
	Percentage of population with access to gas		%	0.015929		Statistical data ^a^
	Engel’s coefficient (Urban)		%	0.016347	√	Statistical data ^a^
	Engel’s coefficient (Rural)		%	0.015804		Statistical data ^a^
	Percentage of graduates in junior secondary school entering senior secondary school		%	0.016032	√	Statistical data ^a^
	Popularity rate of environmental propaganda and education		%	0.016281	√	Statistical data ^a^
	Number of public transportation vehicles per 10,000 persons		unit/10,000 persons	0.013470		Statistical data ^a^
	R&D expenditures as percentage of GDP		%	0.013579		Statistical data ^a^
Ecology	Coverage rate of forest	Mountain	%	0.013158		Eco-city planning ^c^
		Plain		0.016929	√	Eco-city planning ^c^
		Seaside		0.017710	√	Eco-city planning ^c^
	Proportion of protected area to land area		%	0.016032	√	Eco-city planning ^c^
	Proportion of nature reserve area to land area		km^2^	0.016261	√	Eco-city planning ^c^
	*Per capita* public green areas		m^2^/person	0.016599	√	Eco-city planning ^c^
	Coverage rate of afforestation in developed areas		%	0.013419		Eco-city planning ^c^
	Coverage rate of green areas in developed areas		%	0.01674	√	Eco-city planning ^c^
	Recovery rate of degraded land		%	0.016846	√	Eco-city planning ^c^
	Overdraft rate of groundwater		%	0.018539	√	Eco-city planning ^c^
	Coverage of wetland		%	0.015894	√	Eco-city planning ^c^
	Tourism area quality rate		%	0.016132	√	Eco-city planning ^c^
Environment	Ambient air quality fine rate		%	0.014321		Report of environmental quality ^b^
	Inshore area water quality rate		%	0.016492	√	Report of environmental quality ^b^
	Coverage of noise control districts		%	0.01453		Report of environmental quality ^b^
	COD emission of GDP		kg/$	0.019699	√	Report of environmental quality ^b^
	SO_2_ emission of GDP		kg/$	0.019951	√	Report of environmental quality ^b^
	COD emission of value-added industry		Kg/$	0.021234	√	Report of environmental quality ^b^
	Carbon emission of GDP		kg/$	0.016857	√	Statistical data ^a^
	Rate of reaching the standard of industrial waste water		%	0.016149	√	Eco-city planning ^c^
	Rate of industrial dust removal		%	0.014313		Eco-city planning ^c^
	Urban sewage treatment rate		%	0.016353	√	Eco-city planning ^c^
	Rate of sewage disposal		%	0.014221		Statistical data ^a^
	Industrial water recycling rate		%	0.016616	√	Statistical data ^a^
	Innocuous disposal rate of garbage		%	0.013814		Eco-city planning ^c^
	Disposal rate of industrial waste residue		%	0.013643		Statistical data ^a^
	Rate of comprehensive usage of industrial waste residue		%	0.015072		Eco-city planning ^c^
	Urban drinking water sources quality rate		%	0.016132	√	Report of environmental quality ^b^
	Drinking water quality rate		%	0.016132	√	Report of environmental quality ^b^
	Comprehensive index of air environmental quality		/	0.019538	√	Report of environmental quality ^b^
	Final consumption of energy		ton SCE	0.016585	√	Statistical data ^a^
	Qualified rate of executing “Three Meaning”		%	0.014132		Statistical data ^a^
	Collection of discharge fee		$	0.014657		Statistical data ^a^
	Proportion of environmental protection investment to GDP		%	0.016705	√	Statistical data ^a^

^a^ Statistical yearbook of Tianjin, 2006–2009. ^b^ Report of environmental quality of Tianjin, 2005–2008. ^c^ Eco-city planning of Tianjin.

**Table 4:** The DPSIR framework with the final weights and ecological security scores.

Dimension of the indicator	Macro-indicators		Indicator name	Specific indicators		The thresholds of urban ecological security	
	The weight of systems	The ecological security scores for 2008		The weight of indicators	The eco-security scores in 2008		
						Unsafe	Safe
Driving force	0.1989	0.1764	*Per capita* disposable income of urban households	0.2254	0.2254	5,000	18,000
			**Proportion of actual investment of protecting the environment in projects**	0.3155	0.3155	3	8
			**Percentage of graduates in junior secondary schools entering senior secondary school**	0.2511	0.2511	70	90
			Value added of industry *per capita*	0.2178	0.1002	12	50
Pressure	0.2304	0.1717	Energy consumption of GDP	0.0788	0.0538	1.5	0.6
			Water consumption of GDP	0.0788	0.1012	150	50
			Ratio of clean energy consumption	0.0894	0.0833	20	30
			**Fresh water consumption of value added in industry**	0.1305	0.11223	30	9
			Energy consumption of value added in industry	0.0894	0.0384	1.8	0.4
			Population density	0.0743	0.0639	2000	500
			Overdraft rate of groundwater	0.0875	0.0585	4	1.5
			COD emission of GDP	0.0841	0.0895	6	1.5
			SO_2_ emission of GDP	0.0955	0.0654	7	1
			**COD emission of value added of industry**	0.1129	0.1129	10	5
			Carbon emission of GDP	0.0788	0.0538	3,750	1,500
State	0.1877	0.1135	Coverage rate of forest (mountain)	0.1044	0.0448	18	25
			Coverage rate of forest (seaside)	0.1022	0.0511	7	10
			Proportion of protected area to land area	0.1144	0	17	25
			Proportion of nature reserve area to land area	0.1141	0.0955	4	15
			Coverage rate of green areas in developed areas	0.1029	0.0751	10	40
			Coverage of wetland	0.1131	0.1074	8	15
			Tourism area quality rate	0.1150	0.1155	80	90
			**Inshore area water quality rate**	0.1158	0	80	90
			**Urban drinking water sources quality rate**	0.1155	0.1155	80	95
Impact	0.1900	0.0630	Rate of public satisfaction with environment	0.1640	0.1640	80	90
			*Per capita* public green areas	0.1571	0.04870	5	18
			**Comprehensive index of air quality**	0.2017	0.2017	1.5	1
			Final consumption of energy	0.1446	0.06940	5,500	4,800
			Engel’s coefficient (urban)	0.1622	0.1622	40	60
			**Drinking water quality rate**	0.1690	0.1690	80	95
Response	0.1930	0.0712	Coefficient of effective utilization of agriculture irrigation water	0.1128	0.0221	0.3	0.6
			Popularity rate of central heating	0.1109	0.0743	65	95
			Perfectness ratio of lifeline systems in the city	0.1095	0.0361	80	95
			Popularity rate of environmental propaganda and education	0.1126	0.1126	60	90
			Recovery rate of degraded land	0.1104	0.0662	75	90
			**Rate of reaching the standard of industrial waste water**	0.1135	0.1135	80	95
			Urban sewage treatment rate	0.1058	0.1058	60	80
			Industrial water recycling rate	0.1084	0.1084	50	80
			**Proportion of environmental protection investment to GDP**	0.1158	0	1.5	3.5

We input the indicator data from 2005–2008 into the GDHAS model to analyze and quantify the impacts of the indicators on the corresponding subsystem and the impacts of the subsystems on the whole urban ecosystem dynamically. The final weights of the subsystems and corresponding indicators were obtained, and the key indicators, with their larger weights in the subsystem, and the key subsystem, with its larger weight in the entire ecosystem, were defined based on the results presented in Table 4. The fuzzy assessment model then was used to determine the ecological security scores of the subsystems and the corresponding indicators for Tianjin City in 2008 based on the data in Table 4. Finally, the total score of the urban ecological security of Tianjin City (0.6001) was calculated using Equation (10) based on the scores of the subsystems presented in Table 4.

### 3.3. Discussion

#### 3.3.1. Comprehensive Assessment

Based on the algorithm of the fuzzy assessment model [11], the level of urban ecological security can be divided into the following five ranks: high risk, low risk, alert level, low safety, high safety (Table 5). According to the results presented in Section 3.2, the total score of the urban ecological security of Tianjin City in 2008 was 0.5958. This value is in the range of alert level and shows that the status of urban ecological security of Tianjin City is not very stable. Tianjin city is an important industrial city of China, and industrial pollution pollutant emissions lead to significant environmental pollution of water, air and soil. Meanwhile the urbanization of Tianjin city become faster and faster in recent years, pollution derived from building construction is on the rise and the heat island effect of this city is worsen. Although the measures of controlling the ecological damage and environmental pollution have been enhanced recently, the eco-environmental quality of Tianjin is still relatively poor, and the interference of human beings will lead to the deterioration of the urban ecosystem.

**Table 5:** Levels of urban ecological security.

Ecological security rank	I	II	III	IV	V
Explanation	Unsafe		Critical	Safe	
	High risk	Low risk	Alert level	Low safety	High safety
Range of scores	0–0.2	0.2–0.4	0.4–0.6	0.6–0.8	0.8–1

The levels of the ecological security of the five subsystems can be ordered as follows based on their ecological security scores: driving force > pressure > state > response > impact. The driving force and pressure subsystems are in good condition, but the ecological security levels of the remaining subsystems are relatively low.

The high ecological security score of the driving force subsystem shows that the rapid economic and societal development of Tianjin City offers sufficient financial and personnel resources to protect the eco-environment of the area. The indicators with high ecological security scores, such as the proportion of actual investment for protecting the environment in projects and the percentage of graduates in junior secondary schools entering senior secondary school, play a driving role in the healthy development of the ecosystem of the area. The high ecological security score of the pressure subsystem shows that the intensive usage of resources and energy has increased in conjunction with the development of the economy and society. In addition, the Environment Quality Bulletins of Tianjin city in recent years show industrial pollution (including COD and SO_2_) has been controlled, and the intensity of carbon emissions from industrial source has been reduced, which has lowered the pressure of economic development on the ecological security of Tianjin City.

The ecological security score of the state subsystem is lower (0.1135) than that of the former two subsystems because the long-standing extensive economic development of Tianjin City has caused serious ecological damage and environmental pollution. Apparently, the eco-environmental quality of the area cannot improve in a short time, although the intensive mode of increasing the economy has formed gradually.

The low ecological security score of the response subsystem (0.0712) shows that the measures currently aren’t in place. The measures, including the construction of environmental infrastructures, eco-environmental management, saving resources, and controlling energy consumption and pollution, cannot meet the demand of the social and economic development. As a result, the direct consequences of such measures were the large quantity of pollutants, deterioration of environmental quality and the severe ecological damage, which also would explain the low ecological security score of the impact subsystem.

#### 3.3.2. Key Factors

**Figure 5:**
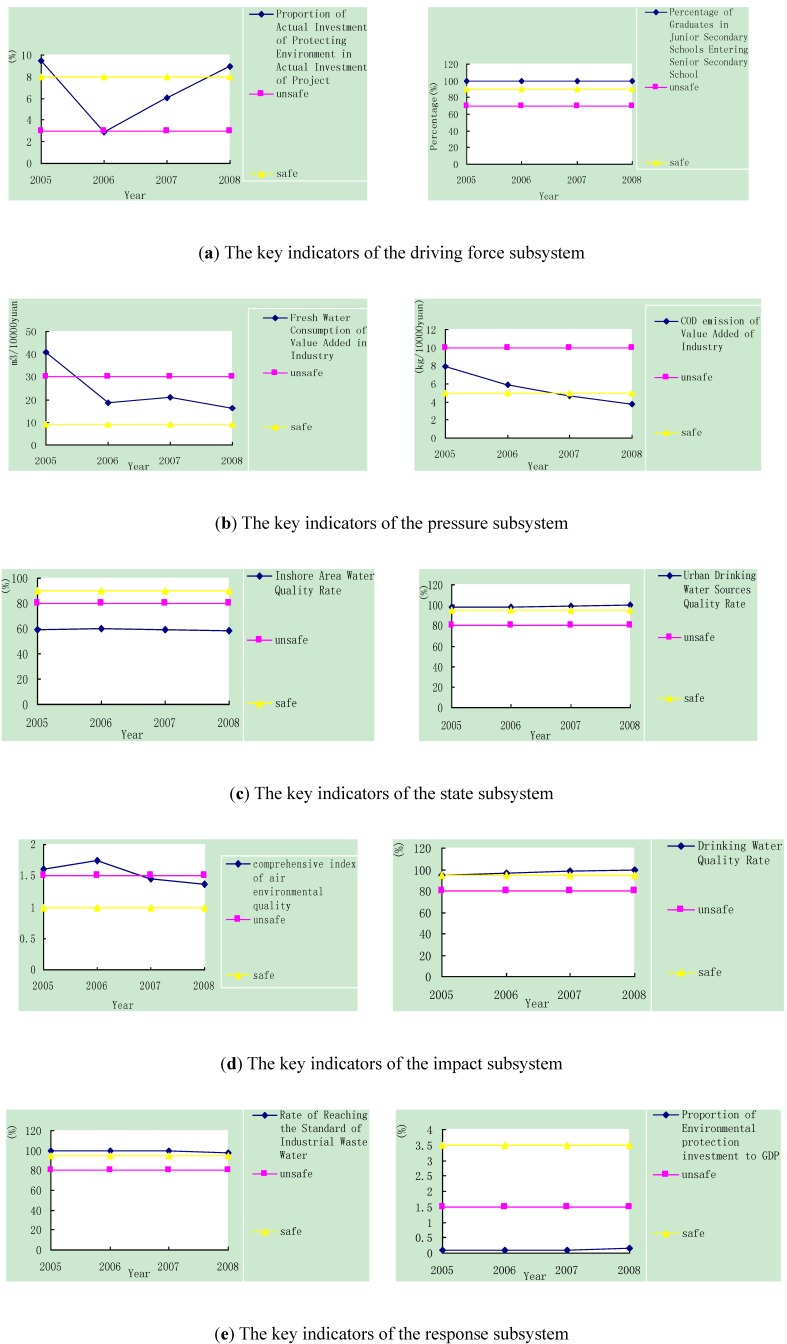
The variation of the key indicators of each subsystem during 2005–2008.

The key subsystem of the DPSIR framework can be defined based on the results shown in Table 4. The pressure subsystem, which had the greatest weight (0.2304) of all five subsystems, can be viewed as the key subsystem of urban ecological security; thus, the pressure subsystem plays a leading role in the urban ecological security system of Tianjin City. Because Tianjin is the crucial industrial base of China, the development of industry leads to the development of the entire city. Thus, reducing the consumption of resources and energy, controlling pollution, and lowering the emission of carbon during the development of industry should be the key measures that will ensure the urban ecological security of Tianjin City. The key indicators of each subsystem were also obtained based on their weights and then analyzed based on their variation during the study period (2005–2008) (Figure 5).

The indicator titled “proportion of actual investment of protecting the environment in projects” is the key indicator of the driving force subsystem, and it can be viewed as the basis of ecological and environmental protection. The value of this indicator dropped to 2.87% below the unsafe level in 2006 but then increased in 2008, reaching 8.9% above the safe level. With the construction of the eco-city of Tianjin, this indicator will continue to increase. The other key indicator of the driving force subsystem, the percentage of graduates in junior secondary schools entering senior secondary school, remained stable and above the safe level during the study period. This result illustrates the driving role of basic education in Tianjin.

In the pressure subsystem, the two key indicators (fresh water consumption of value-added industry and COD emission of value-added industry) reflect the reality that Tianjin lacks fresh water. As Figure 5(b) shows, the first indicator decreased during 2005–2008, and except for 2005, the values were all within the alert level and were approaching the safe level. The second indicator exhibited the same decreasing tendency and reached the safe level in 2008. The decrease of these two indicators shows that with the intensification of industry in Tianjin, the water consumption of industry will further reduced and the emission of industrial wastewater will be further controlled, which in turn will considerably reduce the pressure on urban ecological security.

In the state subsystem, the inshore area water quality rate and the urban drinking water sources quality rate are the key indicators. Because Tianjin is an important coastal city, the water quality of the inshore area is certainly the key factor reflecting the state of the eco-environment. However, with long-standing extensive development, especially around the coastal area, the inshore area water quality rate was below the safe level (Figure 5(c)). The status of urban drinking water is also crucial to a city that lacks water, and this indicator remained above the safe level during the study period. This finding reflects the sound effects of protecting drinking water sources.

Two key indicators of the impact subsystem are the comprehensive index of air quality and the drinking water quality rate. Because coal use plays a significant role in industry and city heating in Tianjin, the impact on air quality by SO_2_ and PM_2.5_ must be taken seriously, and the comprehensive index of air quality should be the key to measure this impact. By controlling industrial air pollution and popularizing central heating, the comprehensive index of air environmental quality decreased during the study period and approached the safe level in 2008 (Figure 5(d)). The drinking water quality rate, which reflects the impact of drinking water safety, has been maintained at a safe and positive trend during the study period (Figure 5(d)) by the contribution of the good condition of drinking water sources.

Finally, the key indicators of the response subsystem are the rate of reaching the standard of industrial waste water and the proportion of environmental protection investment to the GDP, which are related to the measures that directly ensure urban ecological security. The values of the first indicator remained above the safe level during the study period and reached 100% in 2008, which shows that the measures in place to control industrial wastewater worked well. However, the other key indicator remained low and far below the safe level during the study period (Figure 5(e)). This result illustrates that the investment was not enough in environmental protection to ensure the urban ecological security of Tianjin during the study period, and the proportion of investments should further increase to 3.5% which is safe level for urban ecological security.

## 4. Conclusions

The urban ecological security assessment for the purpose of devising an eco-environmental management system is a complicated, multi-hierarchical, multi-factorial, and multi-uncertainty process that should be developed based on the characteristics and development trends of the study area in question. Selecting the representative indicators to build the assessment system plays a crucial role in the entire assessment process. To date, very few studies have used quantitative methods to select indicators before constructing the assessment system.

As human activity rapidly continues to encroach into natural areas, the importance of effective assessment of urban eco-security becomes more imperative. Accurate assessment depends on the selected indicators and quantitative models. Building and empowerment of the index system and fuzzy comprehensive evaluation method are the cores to evaluate urban ecological security, and the threshold states also will affect the accuracy of the evaluation results. In this paper, the GDHAS, an innovative modeling approach based on the traditional AHP approach has been developed; it utilizes the basic method of GTT to select representative indicators according to the characteristics and development trends of a given study area. Indicators selection and quantitative assessment of urban eco-security was carried out, and the comprehensive and dynamical assessment index was set up based DPSIR model. In order to improve the accuracy of the results, the threshold of each index was set on the basis of the relevant standard, statistical result or expert consultations.

The effectiveness of the proposed methodology was verified by applying it to an actual case study: Tianjin City. The results indicate that our systematic methodology is a useful tool for analyzing and assessing the effects of environmental pollution and ecological damage on urban ecological security. The urban ecological security scores for Tianjin City, its subsystems, and their corresponding indicators were obtained and used to evaluate the status and trends of urban ecological security for the entire city. Moreover, the interactions between the subsystems and the indicators can be analyzed through the procedure. The key subsystem of urban ecological security and the key indicators of each subsystem are also defined accurately, which can be used as the basis for decision making in the eco-environmental management of Tianjin City. The proposed methodology also contributes to a significant improvement in the flexible arrangement of the indicators based on the DPSIR framework and GDHAS model, which in turn has enhanced the applicability of the method. If more monitoring data for the indicators were to become available, a more accurate assessment of the urban ecological security can be achieved.
